# Emerging Roles of Neuronal Ca^2+^ Sensor-1 in Cardiac and Neuronal Tissues: A Mini Review

**DOI:** 10.3389/fnmol.2019.00056

**Published:** 2019-03-04

**Authors:** Tomoe Y. Nakamura, Shu Nakao, Shigeo Wakabayashi

**Affiliations:** ^1^Department of Molecular Physiology, National Cerebral and Cardiovascular Center Research Institute, Suita, Japan; ^2^Department of Biomedical Sciences, College of Life Sciences, Ritsumeikan University, Kusatsu, Japan; ^3^Department of Pharmacology, Osaka Medical College, Takatsuki, Japan

**Keywords:** neuronal calcium sensor-1, frequenin, ion channel, survival, immature heart contraction, hypertrophy, nuclear Ca^2+^ signaling, learning and memory

## Abstract

The EF-hand calcium (Ca^2+^)-binding protein, neuronal Ca^2+^ sensor-1 (NCS-1/frequenin), is predominantly expressed in neuronal tissues and plays a crucial role in neuronal functions, including synaptic transmission and plasticity. NCS-1 has diverse functional roles, as elucidated in the past 15 years, which include the regulation of phosphatidylinositol 4-kinase IIIβ (PI-4K-β) and several ion channels such as voltage-gated K^+^ and Ca^2+^ channels, the D2 dopamine receptors, and inositol 1,4,5-trisphosphate receptors (InsP_3_Rs). Functional analyses demonstrated that NCS-1 enhances exocytosis and neuronal survival after injury, as well as promotes learning and memory in mice. NCS-1 is also expressed in the heart including the Purkinje fibers (PFs) of the conduction system. NCS-1 interacts with K_V_4 K^+^ channels together with dipeptidyl peptidase-like protein-6 (DPP-6), and this macromolecule then composes the transient outward current in PFs and contributes to the repolarization of PF action potential, thus being responsible for idiopathic arrhythmia. Moreover, NCS-1 expression was reported to be significantly high at the immature stage and at hypertrophy in adults. That report demonstrated that NCS-1 positively regulates cardiac contraction in immature hearts by increasing intracellular Ca^2+^ signals through interaction with InsP_3_Rs. With the related signals, NCS-1 activates nuclear Ca^2+^ signals, which would be a mechanism underlying hormone-induced cardiac hypertrophy. Furthermore, NCS-1 contributes to stress tolerance in cardiomyocytes by activating mitochondrial detoxification pathways, with a key role in Ca^2+^-dependent pathways. In this review, we will discuss recent findings supporting the functional significance of NCS-1 in the brain and heart and will address possible underlying molecular mechanisms.

## NCS-1 and its Interacting Proteins

**Figure 1:**
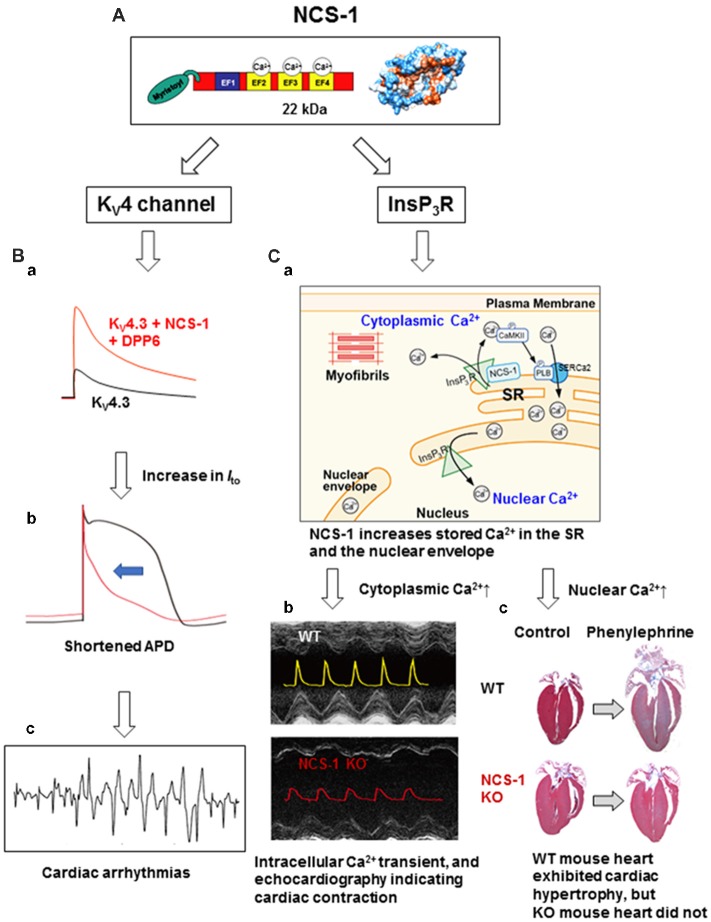
The structure and cardiac functions of NCS-1. **(A)** The structure of NCS-1 (PDB: 1G8I). **(B)** Cartoons demonstrate that NCS-1 and DPP6, both are auxiliary subunits of K_V_4 channels in Purkinje fiber (PF), slows inactivation kinetics of K_V_4 current and increases the current amplitude, respectively, thus increase *I*_to_-mediated K^+^ efflux **(Ba)**. This would accelerate PF repolarization and shortening of APD **(Bb)**, and may lead to cardiac arrhythmias **(Bc)**. The same concepts of the traces in **(B)** were originally reported by Xiao et al. (2013). **(C)** NCS-1 also interacts with IP_3_Rs on the SR and increases local Ca^2+^. This activates CaMKII, followed by CaMKII-dependent phosphorylation of PLB that enhances the Ca^2+^-pump activity of SERCa2, resulting in the increase in SR Ca^2+^ content **(Ca)**. This increases the global Ca^2+^ transient and contraction in the immature heart. NCS-1 deficiency results in a smaller Ca^2+^-transient and contraction (**Cb**; the composite figure of echocardiograms and Ca^2+^ transients are based on data from Nakamura et al., 2011). NCS-1 also increases nuclear Ca^2+^ levels because the SR and the nuclear envelope are interconnected **(Ca)**. NCS-1-mediated increase in nuclear Ca^2+^ signal can promote hormone-induced cardiac hypertrophy, whereas NCS-1 deficiency prevents progression of hypertrophy (**Cc**; adapted from Nakamura et al., 2011). Phenylephrine is an agonist of α1-adrenergic receptor. For further details, please refer to the text. APD, action potential duration; CaMKII, calcium/calmodulin-dependent protein kinase II; DPP6, dipeptidyl peptidase-like protein 6; EF, EF-hand; InsP_3_R, inositol 3,4,5-trisphosphate receptor; *I*_to_, transient outward K^+^ current; KO, knock-out; K_V_, voltage-dependent potassium channel; NCS-1, neuronal Ca^2+^ sensor-1; PLB, phospholamban; SERCa2, sarcoplasmic/endoplasmic reticulum calcium ATPase 2; SR, sarcoplasmic reticulum; WT, wild type.

Intracellular calcium (Ca^2+^) is a versatile second messenger that regulates diverse cellular processes, including neurotransmission, muscle contraction, and signal transduction. Changes in intracellular Ca^2+^ are transduced by multiple proteins, with a key role of a large family of EF-hand Ca^2+^-binding proteins that act as Ca^2+^ sensors or Ca^2+^ buffers. Ca^2+^-buffer proteins chelate Ca^2+^ and often terminate Ca^2+^ signals (Ikura, 1996). In contrast, the binding of Ca^2+^ to Ca^2+^-sensor proteins causes a large conformational change, which consequently transduces the Ca^2+^ signal into various cellular functional changes, by regulating specific target proteins. Calmodulin is one of the best-characterized Ca^2+^-sensor proteins and is involved in many aspects of Ca^2+^ signaling. Neuronal calcium sensor-1 (NCS-1) is the mammalian homolog of the *Drosophila* frequenin protein, which belongs to the larger NCS protein family that includes NCS-1, visinin-like proteins, recoverin, guanylate cyclase-activating proteins, and potassium channel-interacting proteins (KChIPs). The structure of NCS-1 is shown in the Figure 1A, indicating that it is a small (22 kDa) Ca^2+^-binding protein containing 4 EF-hand motifs; of these, 3 (EF2-4) bind to Ca^2+^. Unlike ubiquitously expressed calmodulin, NCS-1 is predominantly expressed in the brain and cardiac tissues, suggesting its specialized roles in these tissues. The Ca^2+^-binding affinity of NCS-1 is significantly higher than that of calmodulin (K_d_ values of ~300 nM vs. ~10 μM, respectively). Although both Ca^2+^-binding proteins can operate within the physiological range of Ca^2+^ levels (~100 nM to ~1–5 μM), the above data suggest that NCS-1 may be more sensitive to small changes in intracellular Ca^2+^.

The functional roles of NCS-1 are still being elucidated. Currently, known functions include the regulation of diverse target proteins, including phosphatidylinositol 4-kinase (PI-4K), voltage- and ligand-gated ion channels, and interleukin-1 receptor accessory protein like-1 (IL1RAPL1). Here, we summarize the current understanding of NCS-1 regulation of some of these target proteins and how it affects brain and cardiac functions (Table 1). Even though NCS-1 has a well-characterized function in these organ systems, it should be pointed out that NCS-1 may have more diverse functions in human physiology and disease, such as a potential role in oncogenesis (Jerng et al., 2004). Such roles should be the focus of future studies.

**Table 1:** Neuronal Ca^2+^ sensor-1 (NCS-1) interacting proteins, known functions of NCS-1 mediated by those proteins and involvement in various diseases.

Interacting proteins	Molecular functions	Physiological roles and involvement in various diseases	References
Phosphatidylinositol 4-kinase IIIβ (PI-4K-β)	Activation	Required for yeast survival	Hendricks et al. (1999)
		Stimulation of exocytosis in neuroendocrine cells	Koizumi et al. (2002), Scalettar et al. (2002), Rajebhosale et al. (2003) and de Barry et al. (2006)
		Facilitation of synaptic transmission in neurons	Taverna et al. (2002) and Zheng et al. (2005)
		Regulation of glucose-induced insulin secretion in pancreatic β cells	Gromada et al. (2005)
		Controlling exocytosis and inflammatory reactions in mast cells	Kapp-Barnea et al. (2003)
Voltage-gated K_V_4 K^+^ channels	Increase in K_V_4-mediated K^+^ efflux	Increasing K_V_4 current amplitude and slowing inactivation time course of A-type current in neurons	Nakamura et al. (2001)
		Regulation of *I*_to_ in cardiomyocytes	Guo et al. (2002) and Nakamura and Coetzee (2008)
		Regulation of PF *I*_to_ and APD together with DPP6 Expression changes result in ventricular arrhythmia	Xiao et al. (2013)
Voltage-gated Ca^2+^ channels	Inhibition (P/Q-type)	Regulation of autocrine pathways in adrenal chromaffin cells	Weiss et al. (2000) and Weiss and Burgoyne (2001)
	Inhibition (N-type)	Reduction of neurite elongation in PC12 cells	Gambino et al. (2007)
	Activation (N-type)	Enhancement of GDNF-induced neurotransmitter release in motoneurons	Wang et al. (2001)
	Activation (P/Q-type)	Activity-dependent synaptic facilitation in nerve terminals	Tsujimoto et al. (2002)
	Activation	Enhancement of neurotransmission and nerve terminal growth in *Drosophila*	Dason et al. (2009)
D2 dopamine receptor	Inhibition of D2 receptor phosphorylation, reduction of the agonist-mediated internalization of the receptor	Preservation of dopamine signaling	Kabbani et al. (2002)
		Promotion of exploration, synaptic plasticity, and rapid acquisition of spatial memory in mice overexpressing NCS-1 in dentate gyrus	Saab et al. (2009)
		Involvement in schizophrenia and bipolar disorder	Koh et al. (2003) and Bai et al. (2004)
		Augmentation of learning and memory in mice	Saab et al. (2009), Mun et al. (2015) and Nakamura et al. (2017)
Inositol 1,4,5-trisphosphate receptors (InsP_3_Rs)	Increase in InsP_3_R channel activity	Enhancement of InsP_3_R-mediated Ca^2+^-signaling	Schlecker et al. (2006)
		Regulation of neurite outgrowth in cultured neurons	Iketani et al. (2009)
		Involvement in long-term depression	Jo et al. (2008)
		Involvement in bipolar disorder	Schlecker et al. (2006)
		Involvement in Taxol-induced Ca^2+^-oscillation and neuropathy	Boehmerle et al. (2006, 2007) and Blachford et al. (2009)
		Promotion of Ca^2+^ signaling and contraction in immature heart	Nakamura et al. (2011)
		Regulation of cardiac hypertrophy	Nakamura et al. (2011)
		Nuclear Ca^2+^ regulation in cardiomyocytes	Nakao et al. (2015)

### Phosphatidylinositol 4-Kinase

#### Role in Exocytosis and Secretion

PI-4K IIIβ (PI-4K-β) catalyzes the synthesis of phosphatidylinositol 4-phosphate, which is a late limiting step in the synthesis of phosphatidylinositol 4,5-bisphosphate, an important lipid regulator of many cellular functions including exocytosis. A yeast homolog of NCS-1 and PI-4-K interact, and NCS-1-induced activation of PI-4-K is required for yeast survival (Hendricks et al., 1999). Structural support for interaction was obtained from a recent NMR structure of Ca^2+^-bound yeast NCS-1 (Ncs1) in complex with an N-terminal yeast PI-4-K (Pik1) fragment (Strahl et al., 2007). This interaction was also detected in neuroendocrine cells (Koizumi et al., 2002; Scalettar et al., 2002; Rajebhosale et al., 2003; de Barry et al., 2006), neurons (Taverna et al., 2002; Zheng et al., 2005) and other cell types including pancreatic beta cells (Gromada et al., 2005) and mast cells (Kapp-Barnea et al., 2003), and was shown to facilitate exocytosis and secretion in these cells (Table 1). However, previous reports have suggested no direct interaction in neurons (Bartlett et al., 2000). This contradiction may be explained by the presence of newly discovered PI-4-Kβ regulators, calneurons. While calneurons interact with PI-4K-β at low Ca^2+^ levels to inhibit its enzyme activity, NCS-1 binds to PI-4K-β at high Ca^2+^ levels to activate it (Mikhaylova et al., 2009), suggesting that calneurons and NCS-1 compete for PI-4-K-β interaction depending on intracellular Ca^2+^ levels. Thus, when the intracellular Ca^2+^ level is low, its interaction might be difficult to be detected.

### Voltage-Gated K_V_4 K^+^ Channels

#### Regulation of Excitability in the Brain

The V7 *Drosophila* mutant that overexpresses NCS-1 has a phenotype of hyperactivity, which results in the proposal that NCS-1 facilitates neurotransmission, possibly by regulating the activities of ion channels (Pongs et al., 1993; Poulain et al., 1994). Indeed, we found that NCS-1 is a Ca^2+^-sensitive regulatory component of a native K^+^ current (Nakamura et al., 2001; Table 1). In the brain and heart, rapidly inactivating (A-type) voltage-gated K^+^ currents control cellular excitability. Although the pore-forming alpha-subunits of these channels are considered to be K_V_4 channels (Serôdio et al., 1994; Fiset et al., 1997; Nakamura et al., 1997), the kinetic properties of K_V_4 channels differ from native A-type currents, suggesting the presence of regulatory subunits. KChIPs, a member of the NCS protein subfamily, were initially reported as a specific K_V_4 regulatory subunit (An et al., 2000). Because NCS-1 modulates K_V_4 currents similar to KChIPs, by increasing current amplitude and slowing the inactivation time course, and NCS-1 physically interacts with K_V_4.2 in mouse brain, it was identified as a regulator of A-type K^+^ currents in neurons (Nakamura et al., 2001; Table 1).

#### Involvement in Cardiac Arrhythmia

This interaction and activation also occurs in adult mouse cardiomyocytes (Guo et al., 2002) and in zebrafish heart (Nakamura and Coetzee, 2008), which lacks KChIPs. The differential regulation of K_V_4 channels by NCS-1 and KChIPs in specific tissues and cell types was an unaddressed topic, and this was clearly demonstrated in the report by Nattel’s group (Xiao et al., 2013). Purkinje fibers (PFs) show an unusual form of transient outward K^+^ current *I*_to_ with slow recovery kinetics and TEA sensitivity compared with ventricular *I*_to_, suggesting a distinct molecular composition. This group found that NCS-1 and DPP6, which were also reported to be auxiliary subunits of K_V_4 K^+^ channels (Jerng et al., 2004), are preferentially enriched in PFs, while KChIP2, an essential subunit of ventricular K_V_4.3 is weakly expressed. Moreover, NCS-1 slowed inactivation kinetics of K_V_4.3, while DPP6 increased its current amplitude, thus increasing the *I*_to_-mediated K^+^ efflux (Figure 1Ba), which would accelerate PF repolarization and shortening of action potentials (Figure 1Bb; similar computer simulation was reported by Xiao et al., 2013). Thus, overexpression of K_V_4 auxiliary subunits may result in steep transmural repolarization gradients in PFs with adjacent ventricular tissues that induces coupled ectopic activity, and potentially leads to lethal arrhythmias (Figure 1Bc). NCS-1 also interacts with the anti-cancer drug taxol (Boehmerle et al., 2006), and is involved in the regulation of taxol-induced cardiac arrhythmia (Zhang et al., 2010). Thus, NCS-1 can be a potential target for anti-arrhythmic therapy.

### Voltage-Gated Ca^2+^ Channels

#### Neurotransmitter Release and Neurite Elongation

NCS-1 regulates voltage-gated Ca^2+^ channels. Published data, however, are somewhat inconsistent with reports demonstrating both positive and negative effects (Table 1). NCS-1 was described to inhibit P/Q-type Ca^2+^ channels an regulates autocrine pathways in adrenal chromaffin cells (Weiss et al., 2000; Weiss and Burgoyne, 2001) and N-type Ca^2+^ channels in PC12 cells, which reduces neurite elongation (Gambino et al., 2007). Other studies, in contrast, have demonstrated positive regulation of N-type Ca^2+^ channels, causing glial cell line-derived neurotrophic factor (GDNF)-induced enhancement of neurotransmitter release in motoneurons (Wang et al., 2001). Activation of P/Q-type Ca^2+^ channels by NCS-1 causes activity-dependent synaptic facilitation in nerve terminals (Tsujimoto et al., 2002). In *Drosophila*, NCS-1 enhances neurotransmission and nerve terminal growth, by functionally interacting with the α1 subunit of the voltage-gated Ca^2+^ channel (Dason et al., 2009). The possible reason for the apparent contradictory findings is that the effects may be cell type-specific and/or mediated by accessory proteins, such as a β-subunit (Rousset et al., 2003), or dependent on other interacting proteins, such as IL1RAPL1 that cooperatively regulates the N-type Ca^2+^ channel *via* NCS-1 (Gambino et al., 2007). In addition, regulation of the Ca^2+^ channel by Ca^2+^ influx through the channel should be considered. Well-characterized examples are Ca^2+^-dependent inactivation (Standen and Stanfield, 1982) and facilitation (Dolphin, 1996) of Ca^2+^ channels regulated by other Ca^2+^-binding proteins, such as calmodulin (Budde et al., 2002; Christel and Lee, 2012). Future research should aim to understand the regulatory mechanisms of Ca^2+^ channels that involve NCS-1.

### D2 Dopamine Receptor

#### Role in Synaptic Plasticity and Psychiatric Illness

Dopamine plays an important role in the reward system of the brain. Disorders of the dopamine system result in several psychiatric and neurological conditions. Dopamine transmission is regulated by dopamine receptor-interacting proteins (DRIP), including NCS-1, calcyon, and DARPP-32. NCS-1 directly interacts with the D2 dopamine receptor, inhibits D2 receptor phosphorylation, and reduces the agonist-mediated internalization of the receptor (Kabbani et al., 2002), indicating that NCS-1 preserves dopamine signaling (Table 1). Indeed, modest NCS-1 overexpression in the dentate gyrus in mice promotes exploration, synaptic plasticity, and rapid acquisition of spatial memory (Saab et al., 2009). NCS-1 is upregulated in the prefrontal cortex of patients with schizophrenia and bipolar disorder (Koh et al., 2003; Bai et al., 2004). Because the levels of other DRIPs were also changed in patients with schizophrenia (Bai et al., 2004; Souza et al., 2008), DRIP signaling is possibly involved in psychiatric disorders. Furthermore, recent findings indicate the N-terminal 60 residues of NCS-1 are responsible for binding to the D2 receptor (Woll et al., 2011). Such knowledge would provide an opportunity to screen for drugs that can specifically interrupt the NCS-1-D2 dopamine receptor interaction and thus prevent psychiatric disorders.

#### Role in Learning and Memory and Possible Mechanism

Several studies have demonstrated that NCS-1 modulates learning and memory. For example, deletion or reduction of NCS-1 resulted in dysfunction of learning and memory in *Caenorhabditis elegans* (Gomez et al., 2001), as well as in mice (Mun et al., 2015), whereas mice overexpressing NCS-1 rapidly acquire spatial memory (Saab et al., 2009). Thus, NCS-1 affects neurophysiology, possibly through various interacting proteins. A mechanism underlying NCS-1-mediated learning and memory was further investigated (Nakamura et al., 2017). *Ncs1*^−/−^ mice exhibited impaired spatial learning and memory function in the Morris Water Maze test, with slight changes in their exercise activity or a structural change in the hippocampus. However, the levels of brain-derived neurotrophic factor (BDNF), a key regulator of memory function, and dopamine were decreased. Furthermore, phosphorylation of Ca^2+^/calmodulin-dependent protein kinase II-α (CaMKII-α), which regulates long-term potentiation, and BDNF levels were decreased, suggesting that CaMKII-α signaling that increases BDNF production is at least partly involved in NCS-1-mediated learning and memory function.

### Inositol 1,4,5-Trisphosphate Receptors

#### Role in Neuronal Pathogenesis

Ca^2+^ signaling *via* inositol 1,4,5-trisphosphate receptors (InsP_3_Rs) regulates cellular function and is involved in pathogenesis (Table 1). NCS-1 physically interacts with InsP_3_R1 and enhances InsP_3_R-mediated Ca^2+^ signaling in rat brains. Indeed, physical/functional interaction of these proteins was directly demonstrated in an *in vitro* experiment showing that the addition of NCS-1 to InsP_3_R1 in the lipid bilayer increased InsP_3_R channel activity (Schlecker et al., 2006). This interaction was also detected at the growth cone region of neurites in cultured neurons, and indicated to be crucial for neurite outgrowth (Iketani et al., 2009). Metabotropic glutamate receptor-mediated cis also mediated by NCS-1/InsP_3_R interaction (Jo et al., 2008). Pathologically, NCS-1/InsP_3_R1 interaction is believed to be involved in bipolar disorder (Schlecker et al., 2006) because lithium, a medical drug for bipolar disorder, inhibited the NCS-1-induced enhancement of InsP_3_R function. NCS-1/InsP_3_R interaction is also considered to mediate neuropathy (Boehmerle et al., 2006, 2007; Blachford et al., 2009), as paclitaxel (taxol), a chemotherapeutic agent used for the treatment of solid cancers, modulates the expression/function of NCS-1, and hence InsP_3_R1-mediated Ca^2+^ signaling.

#### Enhancement of Immature Heart Contraction and Hypertrophy

NCS-1/InsP_3_R interaction is also detected in the heart and is crucial for contraction at the immature stage and cardiac hypertrophy in adult (Nakamura et al., 2011; Table 1 and Figure 1C). A high expression of NCS-1 was found in the immature heart (Nakamura et al., 2003, 2011), but its function at this stage was unknown. Using *Ncs1^−/−^* mice, Nakamura et al demonstrated that NCS-1 contributes to an increase in contraction and Ca^2+^ signaling, specifically at the immature stage (Figure 1Cb). Intracellular Ca^2+^ levels and the sarcoplasmic reticulum (SR) Ca^2+^ content were significantly lower in *Ncs1*^−/−^ myocytes at the neonatal stage than in wild-type cells. Mechanistically, the interaction of NCS-1 with InsP_3_R increases InsP_3_R-dependent Ca^2+^ signaling, followed by the activation of CaMKII-dependent pathways, and promotes SR Ca^2+^ pump *via* the phosphorylation of phospholamban (PLB), which ultimately induce increase in the SR Ca^2+^ content and global Ca^2+^ transient, thus cardiomyocyte contraction (Figures 1Ca,b). The importance of crosstalk among NCS-1, InsP_3_Rs, and CaMKII in the immature hearts was evident by the high expression of all three proteins in immature hearts (Nakamura et al., 2011). In the neonatal mouse heart, the structure and function of SR are immature. Nonetheless, it is considered a primary source of Ca^2+^ necessary for muscle contractions, suggesting the existence of factors missing during development and promoting SR-dependent excitation-contraction (E-C) coupling in the postnatal stages. NCS-1 may act as one of these missing factors. Numbers of molecules which levels are high at the immature stage are often up-regulated in the disease conditions, such as cardiac hypertrophy. NCS-1 is also highly expressed during the early stages of hypertrophy in the adult heart and promotes the progression of hypertrophy, at least in part, through InsP_3_R activation (Nakamura et al., 2011; Figure 1Cc). A possible molecular mechanism is suggested in the next section.

#### Regulation of Nuclear Ca^2+^ Signals

The aforementioned data indicate that NCS-1 can discretely regulate different types of Ca^2+^ signaling pathways in the heart (i.e., regulation of E-C coupling in immature heart and changes in gene expression in the adult heart). Recent evidence suggests that gene transcription is regulated by nuclear Ca^2+^ signals. However, the mechanisms underlying nuclear Ca^2+^ regulation and its relationship to cytoplasmic Ca^2+^ regulation have not been completely solved. Using a subcellular-specific, fluorescent protein-based Ca^2+^ indicator GECO (Zhao et al., 2011; Nakao et al., 2015) confirmed the following: (1) nuclear Ca^2+^ transients were elicited by both electrical and receptor stimulations (with insulin-like growth factor-1, IGF-1) in neonatal mouse ventricular myocytes; and (2) receptor stimulation-elicited nuclear Ca^2+^ transients were mainly mediated by InsP_3_Rs. Furthermore, based on the evidence that IGF-1-elicited nuclear Ca^2+^ transient was significantly diminished in *Ncs1*^−/−^ cardiomyocytes, NCS-1 is involved in the receptor stimulation-induced nuclear Ca^2+^ regulation through interaction with InsP_3_Rs (Nakao et al., 2015; Table 1). This may contribute to NCS-1-mediated hypertrophy, which was described above. A possible mechanism underlying a dual effect of NCS-1 on nuclear Ca^2+^ signals and E-C coupling is that NCS-1 increases the Ca^2+^ content of SR (Nakamura et al., 2011) that is interconnected to the nuclear envelope (Wu and Bers, 2006) and consequently may increase nuclear Ca^2+^ (Nakao et al., 2015; Figure 1Ca).

### Other Functions of NCS-1 With Unknown Interacting Proteins

#### Enhancement of Neuronal Survival After Injury

Physical or chemical injury and genetic abnormalities can result in neuronal degeneration, which may underlie human neurodegenerative disorders, such as Alzheimer’s disease and Parkinson’s disease. Both intrinsic and extrinsic factors, including neurotrophic factors, can activate the anti-apoptotic process to rescue neuronal cell death. The signaling pathway leading to cell survival remains unresolved. In this regard, NCS-1 was found to be a novel Ca^2+^-dependent survival-promoting factor upregulated in injured neurons (Nakamura et al., 2006), based on the following observations. (1) NCS-1 expression increases in injured neurons; (2) NCS-1 overexpression diminished various stress-induced neuronal cell death in culture; and (3) the dominant negative EF-hand NCS-1 mutant (E120Q) accelerated cell death. Mechanistically, the expression level of NCS-1 in neuron is increased by GDNF, a neurotrophic factor upregulated by neuronal injury, and NCS-1 mediates GDNF survival signaling *via* the activation of the Akt pathway.

#### Role in Stress Tolerance in Cardiomyocytes

Not only in neurons, NCS-1 also plays a key role in protecting cardiomyocytes against stress through the activation of mitochondrial detoxification pathways (Nakamura et al., 2016). Excessive stress induces cytosolic Ca^2+^ overload and cell death. In contrast, mild forms of stress lead to physiologically relevant changes in Ca^2+^, which activate Ca^2+^-dependent survival pathways by binding to Ca^2+^-sensor proteins. As one such protein, NCS-1 was found to play important roles in Ca^2+^-dependent survival signaling. *Ncs1^−/−^* myocytes were more susceptible to oxidative and metabolic stress, and cellular ATP levels, mitochondrial respiration and biosynthesis were significantly reduced in these cells. In wild-type myocytes, mild oxidative stress increased the mitochondrial proton leak, which exerted a protective effect by inhibiting the production of reactive oxygen species. However, this response was diminished in *Ncs1*^−/−^ cardiomyocytes, thus resulting in cell death. Similar susceptibility was also observed in *Ncs1*^−/−^ hearts subjected to ischemia-reperfusion injury. In these hearts, molecules regulating Ca^2+^-dependent survival pathways, such as Akt and PGC-1α, which promote mitochondrial biogenesis and function, were significantly downregulated compared to wild-type hearts. These data demonstrate a novel role of NCS-1 that contributes to stress tolerance in cardiomyocytes, partly by the activation of Ca^2+^-dependent survival pathways. NCS-1 may also participate in cardioprotection by mediating receptor-signaling pathways. For example, NCS-1 associates with, and modulates, adenosine receptor activity (Navarro et al., 2012). Given the central role of adenosine in mediating the protective effects of ischemic preconditioning (Cohen and Downey, 2008), it is entirely possible that the cardioprotective effects of NCS-1 is partially mediated by this pathway.

## Conclusion

Recently, studies have elucidated new roles of NCS-1 in physiology and pathophysiology. In this review, we have mainly focused on NCS-1 in the neuronal system and heart. Our particular interest is the emerging theme that NCS-1 directly regulates the function of several ion channels that permeate Ca^2+^ (e.g., several types of voltage-gated Ca^2+^ channels, ionotropic dopamine receptors, and InsP_3_Rs), suggesting a general role of Ca^2+^ influx *via* the channel that binds to NCS-1 and consequently regulates channel functions and/or downstream Ca^2+^-dependent signaling, which affect various neuronal and cardiac functions. Furthermore, many established roles of NCS-1 are related to protective responses of cells against exogenous stress that leads to mild increases in cytosolic Ca^2+^. This suggest that intracellular Ca^2+^ as a determinant of cell survival and cell death, and Ca^2+^-sensor proteins, such as NCS-1, may serve as a switch to proceed the signal. We believe that this review provides intriguing observations and compels researchers to conduct detailed investigations and extend their studies on NCS-1 and its important regulatory proteins.

## Author Contributions

TN conducted most part of research, wrote, organized, and finalized the article. SN did some experiments and wrote some part of the article. SW contributed to the discussion on all part of the study and wrote some part of the manuscript. SN and SW wrote and edited some parts of the article.

## Conflict of Interest Statement

The authors declare that the research was conducted in the absence of any commercial or financial relationships that could be construed as a potential conflict of interest.
